# The Basic Psychological Needs in the Classroom Scale (BPN-CS)

**DOI:** 10.3390/bs11070096

**Published:** 2021-06-24

**Authors:** Pedro Javier Conesa, Jon Andoni Duñabeitia

**Affiliations:** 1 Facultad de Educación, Universidad de Murcia, 30100 Murcia, Spain; pj.conesacervera@um.es; 2 Centro de Investigación Nebrija en Cognición, Universidad Antonio de Nebrija, 28015 Madrid, Spain; 3 Department of Language and Culture, The Arctic University of Norway, 6050 Tromsø, Norway

**Keywords:** BPN-CS, basic psychological needs, self-determination theory, measurement, elementary school, validation

## Abstract

Recent experimental and theoretical studies have shown that the assessment of students’ satisfaction of their basic psychological needs (*BPN*) can be a powerful resource to identify different areas to improve their well-being, engagement, or learning achievement in school contexts. However, currently, the number of validated tools to assess the satisfaction of the BPN is very low, hindering informed decision-making strategies at the educational level. The aim of this study was to develop and validate the Basic Psychological Needs in the Classroom Scale (BPN-CS) instrument, adapting existing instruments and putting the new tool to the test. The BPN-CS was developed to measure the level of satisfaction of autonomy, competence, relatedness, and novelty in the classroom. We tested the scale on a representative sample of 1344 Spanish elementary school students from 8 to 13 years old. A series of analyses were run in order to test the internal consistency of the main factors as well as to prove the convergent and divergent validity of the instrument. In summary, the BPN-CS is presented as a reliable and valid self-report instrument to measure basic psychological needs in a classroom context with elementary school pupils in the Spanish context.

## 1. Introduction

The self-determination theory (SDT) [1,2] stems from the premise that all individuals have a natural tendency toward growth and healthy development. The fundamental motivational energy that makes individuals grow and develop comes from a set of basic psychological needs that are crucial for a person to experiment, endure, and promote welfare, internalization, and learning. This idea about the satisfaction of a set of psychological needs yielding growth and improvement is the core premise of the basic psychological needs theory (BPNT) [2,3]. Interestingly, the BPNT has been claimed to be relatively universal, extending across ages and cultural origins [4,5].

According to the BPNT, the basic psychological needs are competence, autonomy, and relatedness, which, when satisfied or frustrated, show a direct effect on an individual’s motivation [6]. The satisfaction or frustration of these needs depend on multiple internal and external (contextual) factors [7,8], and while both their satisfaction and frustration are closely interrelated constructs, the former has been classically associated with well-being outcomes, while the latter has been linked to ill-being outcomes [9] While the satisfaction and frustration of basic psychological needs do not necessarily represent the exact same concepts and produce the same outcomes, the SDT literature has typically measured satisfaction as a composite estimate of both satisfaction and reverse-scored frustration items. In this line, in the current study, we focus on need satisfaction as a proxy for general assessment of the basic psychological needs. The need for *competence* is related to the experience of efficacy in a given duty or action. This need reveals itself as the desire to extend an individual’s own capacities and skills [6,10]. The need for *autonomy* refers to the experience of showing will and self-direction within an activity or action, and it represents a critical aspect of behavior regulation [9]. Finally, the need for *relatedness* is conceived as the necessity to establish a successful relationship with others, caring about them and at the same time being cared about [11]. Hence, relatedness is grounded on building safe and close relationships [12]. These three needs are especially relevant in school contexts, since competence is closely related to the perception of success by students when they are involved in learning activities, autonomy is linked to the behavioral regulation that directs students’ actions to an end, and relatedness is grounded in the establishment of positive and supportive social and affective bonds with the peers.

The SDT postulates that the type of regulated motivation that drives students’ experience in school environments is influenced by the satisfaction or frustration of the basic psychological needs [6]. In this sense, the SDT considers different forms of motivational regulation [13] and explains the degree to which behaviors are volitional or self-determined through a motivational continuum. This continuum ranges from the lowest levels of self-determination (*amotivation*) to the highest ones (*intrinsic motivation*), with different intermediate levels (*extrinsic motivation*). An intrinsically motivated learner will participate in an activity for the pleasure and satisfaction it generates. In contrast, students who participate in activities for the effects they obtain from them would be extrinsically motivated [14]. Hence, the least autonomously regulated motivation is the *external* one, and it refers to the performance or behavior oriented at obtaining a reward or avoiding a punishment [15]. If the behavior is performed to avoid guilt or shame, it is understood as a process motivated by *introjected regulation.* When a person chooses to act because the behavior or its outcome is personally important, the process is typically associated with *identified regulation*, as in the case of students who value and accept the benefits associated with the learning activity. Finally, *integrated regulation* occurs when the learner has internalized the reasons for engaging in a behavior. In general terms, and according to the SDT, the satisfaction of psychological needs is associated with increased autonomous regulation (*identified regulation* and *intrinsic motivation*) leading to well-being, adaptation, and optimal functioning [8].

In the last decades, the BPNT has been strongly backed up mainly by theoretical and observational research in the educational field, as well as by experimental research specifically oriented toward certain disciplines, such as physical education [8,10]. As a consequence, there is common agreement that measuring basic psychological needs can be effective to understand the diverse cognitive, social, and motivational aspects of students [7,16]. Different studies conducted in educational contexts show that the satisfaction or frustration of a student’s BPN have a direct impact on wellbeing [17,18,19], intrinsic motivation [20], engagement during class [14,21], or academic achievement [22,23]. Interestingly, this has been shown both for unique needs and for the interaction of all of them in a collaborative way [7].

Recently, several researchers have proposed new candidate needs to be incorporated into the BPNT. One of the more repeatedly suggested ones is the need for *novelty* [24]. The need for novelty is defined as the necessity to experience something not previously known or that differs from the experiences that comprise an individual’s everyday routine [25]. Hence, the experience of novelty has been claimed to be critical for the development of internal motivation, and this is particularly relevant in school contexts. The satisfaction of novelty as a psychological need could be promoted in educational environments by providing students with experiences involving novel activities, tasks, projects, materials, groups, or contacts. Recent studies controlling for the three classic BPN have shown that novelty satisfaction significantly contributes to a person’s motivation by increasing self-reports of vitality, life satisfaction, and general well-being [24]. Nonetheless, and in spite of the great deal of attention received by the novelty dimension in recent years, it is worth noting that its inclusion as a definite BPN is yet to be accepted [10].

In order to measure both the satisfaction and the frustration components of the different elements that are part of the *BPN*, the main tool used is the *Basic psychological need satisfaction and frustration scale* (*BPNSFS*) [5]. This scale was originally created to be used in the adult population, and context-specific adaptations are available including the work-domain [26], sports [27,28,29], or physical education [30,31]. However, a more generalized adaptation to the school context that goes beyond a specific subject or area such as physical education is still to be developed. The school environment represents one of the major life contexts of children. Moreover, given the relevance of the BPN in educational environments and considering the potential usefulness of a valid measuring instrument for school contexts and younger samples, an adapted and validated tool is needed, and the current study aimed at filling this gap by providing the educational community with an adapted and validated scale.

While this is not the first attempt to adapt some instruments from the adult scale and test them in school contexts, it is worth underlining that the internal consistency and validity measures for these tools have not been reported. In this line, some researchers have employed differing sub-scales drawn from existing questionnaires to measure the satisfaction of the needs within the school context (see [32]), while others have created totally new instruments such as *The adolescent students’ basic psychological needs at school* scale (ASBPNSS; [33]). This last tool measures the satisfaction of the psychological needs of adolescent students at the school, but it has only been used and tested in a sample of Chinese adolescents, and underlying potential cross-cultural differences may make this instrument unsuited for other samples. Furthermore, it does not include specific items related to the novelty component, which, as stated above, is a critical one in educational contexts.

In the Spanish educational context, and in the absence of other instruments, the most widely used tool is the Spanish physical education version (PE) [34] of the *Basic Psychological Needs in Exercise Scale* (BPNES) [35]. Under the specific context of physical education, several studies have found that fostering a need-supportive teaching style by PE teachers positively affects different types of psychological needs and self-determined motivation [36,37,38]. However, and considering that the BPNES was originally devised as an instrument specifically oriented at physical education contexts, some of the situations proposed in this scale can be hardly generalized to other school contexts, not being transferable to other classroom scenarios (e.g., the item “*Exercise is an activity I do very well*” or the item “*I feel very comfortable when I do exercise with my peers*”). Hence, while the BPNES is an outstanding instrument for PE contexts, an analysis of the items of the scale raises doubts about the generalizability of the results to other classroom settings. Additionally, it should be kept in mind that PE strategies and contexts differ from those associated with many other academic subjects. For example, in many physical education sessions, demonstrations and assessments of competence are often public, whereas, in other academic sessions at school, an individual’s performance may be relatively more covert [39]. In addition, many of the learning goals of PE are qualitatively different from those of other academic subjects, and healthy behaviors (e.g., levels of physical activity or exercise) have been shown to be a possible predictor of satisfaction of basic psychological needs in students in PE contexts [40,41], while transferability to other subjects is yet to be established. Thus, the physical education context represents a clearly different arena for the satisfaction of basic psychological needs, which greatly differs from other classroom settings. Consequently, the use of the same scale may not be suited, and it may be useful to provide a tool that measures the satisfaction of basic psychological needs of elementary students in a broader educational context.

Childhood is an important stage of cognitive and social maturation. Given that the satisfaction of students’ needs at school is closely related to their social and learning outcomes [7,8], it seems important to monitor and ensure the satisfaction of psychological needs at school. Satisfying BPNs has been shown to have significant positive effects on students for their optimal development and functioning [7,23]. As such, it is important to provide the educational community with a scale with adequate psychometric properties that measures the satisfaction of basic psychological needs in elementary school to identify different areas of intervention to improve the well-being, engagement, or achievement of these students. As said, an all-encompassing and validated tool to measure basic psychological needs has not yet been developed for elementary school children. Therefore, the aim of this study was to develop a tool to assess the satisfaction of the basic psychological needs in elementary school students—the *Basic Psychological Needs Satisfaction in the Classroom* Scale (BPN-CS)—by adapting and extending existing scales, and to validate the instrument in a large sample of children. This scale stems from the Spanish physical education version [34] of the BPNES and adapts it to fit the general classroom context. Its psychometric properties were tested among a large sample of Spanish elementary school students. Based on previous studies, the fit of the data to a four-factor model and a general-factor model [42,43] was explored. The internal consistency of its subscales was also investigated, as well as its validity across genders and socioeconomic status.

## 2. Materials and Methods

### 2.1. Participants

In this cross-sectional study, we investigated 1344 elementary school Spanish students from 8 to 13 years old (*M* = 10.3, *SD* = 0.896, 48.5% females). The sample size is similar to that of recent studies following similar approaches in Spanish samples of adolescents (e.g., 1444 participants in [43]). The data collected in this research were part of a larger longitudinal research project (2020–current) aimed at exploring the impact of a world-leading computerized cognitive intervention program (CogniFit Inc., San Francisco, CA, USA) based on the stimulation of the executive functions on the motivation of the studentship. The sample included students of 26 public and private Spanish schools, in the last three years of Elementary Education (fourth, fifth, and sixth grades). A total of 27.5% of the students were enrolled in fourth grade, 40.3% in fifth grade, and 32.2% in sixth grade.

### 2.2. Measure

*The Basic Psychological Needs Satisfaction in the Classroom Scale* (BPN-CS; see Appendix A) was created by adapting to the elementary classroom context the Spanish version by [34] of the *Basic Psychological Needs Exercise Scale* (BPNES; [35]). The adaptation included 17 items grouped into four subscales: autonomy satisfaction (e.g., “*I feel I have been doing what really interests me in class*”), competence satisfaction (e.g., “*In class, I feel confident that I can do things well”*), relatedness satisfaction (e.g., “*I feel like I have a close relationship with my teachers and classmates*”), and novelty satisfaction (e.g., “*I frequently feel there are novelties for me in the classroom*”). Responses were collected by asking students to select one out of five values in a 5-point Likert-type scale from 1 (strongly disagree) to 5 (strongly agree). The five items related to the novelty satisfaction variable were taken and adapted from the *Novelty Need Satisfaction and Frustration Scale* (NNSFS; [25]).

### 2.3. Procedure

This study procedure was approved by the University of Murcia’s Research Ethics Commission (Ref: 2989/2020). Since the sample was composed of underage participants, parents were requested to sign a parental consent form. Participants completed a 20-min online study programmed using the Gorilla Experiment Builder [44]. Project staff contacted the schools and asked them to participate in the study. After the schools’ staff gave their consent to participate in the study, we initiated an information session for parents and teachers, in which the child and his or her parent volunteered to participate. Students were informed that their responses would be confidential and used only for research purposes. Participation in the study was voluntary and participants could give up at any time.

### 2.4. Statistical Analysis

Descriptive statistics, Pearson’s correlations, reliability analysis, and confirmatory factor analysis were conducted with JASP v.14.1 software using the SEM module (*lavaan package*). Firstly, a descriptive analysis of the 17 items in the questionnaire was carried out, considering the means and standard deviations, skewness, and kurtosis. The assumption of univariate normality was examined by standardized values for univariate skewness and kurtosis coefficients [45]. The *t*-test was used to analyze gender differences between need satisfaction. Following this, a correlation analysis was performed for the four-factor model of the BPN-CS test. The Cronbach’s alpha coefficient and Mc Donald’s omega were used to analyze the reliability of the scale. In comparison with Cronbach’s alpha, McDonald’s omega has the advantage of taking into account the strength of association between items and constructs as well as item-specific measurement errors [46]. The Kaiser–Meyer–Olkin sample adequacy measure (*KMO*) and the Bartlett sphericity test were used to test the adequacy of the sample to the factor analysis. Next, a confirmatory factor analysis (*CFA*) was performed using a robust weighted least square (*DWLS*) estimator. *DWLS* was chosen because this estimator is more suitable due to the ordered–categorical nature of Likert scales, rather than the traditional maximum likelihood estimation [47,48], resulting in more precise estimates of key model-parameters. The factor loadings were analyzed using three subscales proposed by Chen et al. [5] in addition to the proposal of González-Cutre et al. [24], including novelty as the fourth basic psychological need. The goodness of fit was judged with the following fit indexes: the ratio between the Chi-squared value and the degree of freedom (*X*^2^/*df*), the comparative fit index (*CFI*), the Tucker–Lewis Index (*TLI*), the standardized root–mean–square residual (*SRMR*), the root–mean–square error of approximation (*RMSEA*), and with a confidence interval at 90% (*90% CI*). After this, and in order to explore measurement invariance across gender (group 1 = girls, group 2 = boys) and socioeconomic status, a multiple-group confirmatory factor analysis (*MGCFA*) was performed. The measurement invariance types considered in this study were a configural model, metric, scalar, and strict invariance. The configural invariance indicates that participants from different groups conceptualize the constructs in the same way. The metric invariance tests if different groups respond to the same factor loadings. The scalar invariance compares latent means, and the strict invariance is the equivalent of the residual items [49]. An *MGCFA* following these steps is widely accepted as the best approach for testing measurement invariance [45]. In this study, according to Chen [50], we used criteria of a −0.01 change in CFI, paired with changes in RMSEA of 0.020 and SRMR of 0.030 for metric invariance and 0.010 for scalar invariance.

## 3. Results

### 3.1. Descriptive Analysis

A detailed sample description is presented in Table 1. The means, standard deviations, skewness, and kurtosis of the four subscales of the 17-item BPN-CS are presented split by gender in Table 2. No missing values were observed. Standardized values ranged from −1.37 to −0.11 for skewness and from −0.32 to 1.76 for kurtosis, sustaining the assumption of univariate normality [48].

In order to check the adequacy of the sample to the factor analysis, the Kaiser–Meyer–Olkin sample adequacy measure (*KMO*) and the Bartlett sphericity test were used. The *KMO* index showed a good value of 0.90 and the values of the Bartlett sphericity test were statistically significant (*X*^2^ = 7538; *p* < 0.001). The internal consistency of the scale was evaluated with two indexes: Cronbach’s α and McDonald’s ω [51]. The results showed good internal consistency in all subscales. The measures associated with the reliability of the subscales are presented in Table 3.

Correlations between the satisfaction of the four main psychological needs are presented in Table 4. As expected, all the subscales were strongly and positively correlated with each other.

### 3.2. Confirmatory Factor Analysis

The factorial loadings of the individual items of the four sub-scales of the 17-item BPN-CS are presented in Table 5. Factor loadings of all items were statistically significant within their respective factor, and factor loadings ranged from 0.57 (*Q*16) to 0.81 (*Q*9) (girls: factor loadings ranging from 0.57 (*Q*3) to 0.84 (*Q*13); boys: factor loadings ranging from 0.53 (*Q*10) to 0.84 (*Q*9)).

In order to find the most optimal model, the above proposed four-factor model (autonomy satisfaction with the items Q1, Q5, Q9, and Q14; competence satisfaction with the items Q2, Q6, Q10, and Q15: relatedness satisfaction with the items Q3, Q7, Q12, and Q16; novelty satisfaction with the items Q4, Q8, Q11, Q13, and Q17) was compared with a general factor including the seventeen items (see Table 6). The four-factor model showed an excellent fit for the data (*X*^2^ = 222.06, *p* < 0.001, *X*^2^/*df* = 1.96, *CFI* = 0.99, *TLI* = 0.99, *SRMR* = 0.04, *RMSEA* = 0.03). In contrast, the model with a general factor showed a much poorer fit (*X*^2^ = 771.11, *p* < 0.001, *X*^2^/*df* = 6.47, *CFI* = 0.95, *TLI* = 0.94, *SRMR* = 0.08, *RMSEA* = 0.06).

### 3.3. Multiple-Group Confirmatory Factor Analysis

A multiple-group approach was used to test measurement invariance across gender. Configural invariance criteria were met, as noted by the models’ good fit indices across gender (see model 1 in Table 7). Criteria for metric invariance (model 2) was also met, showing that the BPN-CS was invariant across gender groups (Δ*CFI*  =  − 0.002; *ΔRMSEA*  =  0.003). The results showed that the criteria for scalar invariance (model 3) were fully met across gender groups (Δ*CFI*  =  0.000; Δ*RMSEA* = −0.001). Criteria for strict invariance (model 4) were also met across gender groups (Δ*CFI * =  0.000; Δ*RMSEA * =  −0.002).

## 4. Discussion

The main objective of this study was to adapt the Spanish physical education version [34] of the *BPNES* [35] to the general classroom context and to validate it in a large sample of elementary school students. Additionally, this study aimed at validating an instrument that could also account for the satisfaction of the need for novelty, which is a critical factor recently highlighted in the literature [24,25]. To this end, a new instrument was developed by adapting and extending an existing tool: *The Basic Psychological Needs Satisfaction in the Classroom* Scale (BPN-CS). The tool was then applied to a large and heterogeneous group of children, and the results were analyzed following classic scale validation approaches (including descriptive and factorial analysis). The results supported the validity of this instrument, as has been previously the case for other similar studies with different adaptations of the original scale for different samples [43,52,53,54,55,56].

Based on previous studies, two models were tested in a confirmatory factor analysis (*CFA*): a four-factor model and a general-factor model. The results of this *CFA* confirmed the four-factor structure as a good fitting model for the *BPN-CS*, confirming once again the assumption of the SDT that the satisfaction of the different psychological needs (including the recently incorporated need for novelty [24,25]) is best explored and represented as different constructs [6]. Additionally, regarding the reliability of the scale, the internal consistency analysis provided an adequate value for the Cronbach’s alpha and Mc Donald’s omega (ranging from 0.72 to 0.78) in each of the four subscales. These results are similar to those obtained in previous studies [34,42,53], and in line with the findings of [57], where they observed that different cultural context or translation errors do not significantly influence the meaning of the items.

The BPN-CS is a valid instrument that could serve to measure the satisfaction of autonomy, competence, relatedness, and novelty in the classroom. Research literature has observed a direct and indirect relationship in students between the satisfaction of their psychological needs in class and happiness [58], psychological well-being [59], school engagement, or academic achievement [23]. In order to motivate children in the school, it seems essential that they are provided with a class environment in which their basic psychological needs are satisfied. By measuring the level of satisfaction of their students through the use of tools such as the BPN-CS, teachers can get an estimate of their well-being and intervene if deemed appropriate.

Despite the overall positive results obtained in this study, some future directions must be taken into consideration too. Firstly, the sampling technique employed to validate the *BPN-CS* focused on a relatively large Spanish sample of children from different schools. The analysis of the validity and reliability of this very same scale in different cultures and countries could offer a new opportunity to measure these constructs, widening its scope and reach. In addition, future studies should analyze the measurement invariance with respect to other variables, age, academic achievement, or type of center (e.g., public, concerted, or private). In this line, it is worth mentioning that while this was not the main aim of the present study, the large sample size and the relatively balanced gender distribution allowed for a closer look at potential gender differences in the satisfaction of basic psychological needs. No significant differences were observed in the relatedness variable. In contrast, the satisfaction of competence, novelty, and autonomy differed across genders, showing higher scores for girls than boys [60,61] (see Table 2). This observation sets the ground for future studies exploring the underlying mechanisms driving these gender differences.

Furthermore, it is worth mentioning that the BPN-CS exclusively measured the satisfaction, not the frustration, of the needs (similar to the case of the BPNES [34]). The theory of the satisfaction and frustration of the needs with coexistent but separated different constructs is increasingly getting support in the literature [10]. Therefore, the frustration of the needs should be also investigated as an additional set of variables in the future. Additionally, the validity of this new tool could have been explored using similar existing scales. However, it should be kept in mind that existing tools are biased towards specific educational contexts as in the case of physical education (BPNES). Finally, although much research is being done on the need for novelty in recent years, especially in the Spanish context, it should be made clear that novelty is considered a candidate need and is not yet universally accepted as a basic psychological need. Therefore, caution is advised when interpreting this subscale [10].

In sum, this study investigated the psychometric properties of *The Basic Psychological Needs in the Classroom Scale* (BPN-CS) in a Spanish context of elementary school. The results showed that the validity and reliability of the scores derived from the BPN-CS and their subscales (autonomy satisfaction, competence satisfaction, relatedness satisfaction, and novelty satisfaction) were adequate and satisfactory, suggesting that it is a reliable instrument to measure the basic psychological needs described in the self-determination theory in the classroom culture in elementary school students.

## Figures and Tables

**Table 1:** Sample size and demographic characteristics of the participants split by grade.

Grade	*N*	%	Age (Mean)	Age (SD) *	Age (Range)	Girls	%	Boys	%
Total	1344	100	10.29	0.90	8–13	652	48.51	692	51.49
4th grade	370	27.53	9.26	0.48	9–11	183	49.46	187	50.54
5th grade	541	40.25	10.25	0.49	10–12	257	47.50	284	52.50
6th grade	433	32.22	11.21	0.49	10–13	212	48.96	221	51.04

* *Note*. *SD* = standard deviation.

**Table 2:** Descriptive statistics of total sample (*n* = 1344), girls (*n* = 652), and boys (*n* = 692).

	Autonomy Satisfaction			Competence Satisfaction			Relatedness Satisfaction			Novelty Satisfaction		
	Total	Girls	Boys	Total	Girls	Boys	Total	Girls	Boys	Total	Girls	Boys
Mean	3.41	3.50	3.32	4.02	4.06	3.98	4.37	4.39	4.35	3.85	3.91	3.79
*SD* *	0.85	0.84	0.85	0.71	0.71	0.70	0.69	0.70	0.69	0.81	0.80	0.81
Skewness	−0.23	−0.36	−0.11	−0.76	−0.84	−0.68	−1.32	−1.37	−1.28	−0.61	−0.69	−0.55
Kurtosis	−0.29	−0.15	−0.32	0.61	0.70	0.59	1.73	1.76	1.73	0.07	0.20	−0.02
Min	1.00	1.00	1.00	1.00	1.25	1.00	1.25	1.50	1.25	1.00	1.20	1.00
Max	5.00	5.00	5.00	5.00	5.00	5.00	5.00	5.00	5.00	5.00	5.00	5.00

* *Note*. *SD* = standard deviation, Min = minimum, Max = maximum.

**Table 3:** Reliability of the four subscales in the BPN-CS as measured by the internal consistency using Cronbach’s α and McDonald’s ω.

Subscale	Cronbach’s α	McDonald’s ω
Autonomy Satisfaction	0.72	0.72
Competence Satisfaction	0.76	0.76
Relatedness Satisfaction	0.78	0.79
Novelty Satisfaction	0.78	0.77

**Table 4:** Pearson correlation among the satisfaction of basic psychological needs.

	Autonomy Satisfaction	Competence Satisfaction	Relatedness Satisfaction
**Autonomy Satisfaction**	—		
**Competence Satisfaction**	0.58 ***	—	
**Relatedness Satisfaction**	0.42 ***	0.41 ***	—
**Novelty Satisfaction**	0.56 ***	0.44 ***	0.43 ***

*Note.* *** *p* < 0.001.

**Table 5:** Factorial loadings of the BPN-CS by sub-scale and gender.

Factor	Indicator	Total Sample *	Girls *	Boys *
**Autonomy Satisfaction**				
	Q1	0.67	0.66	0.67
	Q5	0.72	0.70	0.73
	Q9	0.81	0.77	0.84
	Q14	0.68	0.70	0.65
**Competence Satisfaction**				
	Q2	0.59	0.61	0.57
	Q6	0.65	0.62	0.68
	Q10	0.59	0.64	0.53
	Q15	0.64	0.63	0.65
**Relatedness Satisfaction**				
	Q3	0.62	0.57	0.66
	Q7	0.60	0.61	0.59
	Q12	0.65	0.68	0.62
	Q16	0.57	0.61	0.54
**Novelty Satisfaction**				
	Q4	0.64	0.63	0.64
	Q8	0.73	0.73	0.74
	Q11	0.75	0.69	0.81
	Q13	0.80	0.84	0.77
	Q17	0.61	0.61	0.60

* Note. All regression weights are significantly different from zero at *p* < 0.001.

**Table 6:** Fit indices for the two models (*n* = 1344).

Model	X^2^	df	X^2^/df	*p*	CFI	TLI	SRMR	RMSEA (90% CI)
Four-factor model	222.06	113	1.96	<0.001	0.99	0.99	0.04	0.03 (0.02–0.03)
General-factor model	771.11	119	6.47	<0.001	0.95	0.94	0.08	0.06 (0.06–0.07)

*Note.* X^2^  =  Chi Squared; df = Degree of Freedom; CFI = Comparative Fit Index; TLI = Tucker–Lewis Index; SRMR = Standardized Root–Mean–Square; RMSEA = Root–mean–square Error of Approximation; 90% CI = 90% Confidence Interval.

**Table 7:** Model fit and measurement invariance between groups according to gender.

Model	X^2^ (df)	X^2^/df	*p*	CFI (ΔCFI)	TLI	SRMR (ΔSRMR)	RMSEA (90% CI) (ΔRMSEA)
Gender							
Model 1: Configural invariance	286.62 (226)	1.26	<0.001	0.995	0.994	0.045	0.020 (0.012–0.027)
Model 2: Metric invariance	325.933 (239)	1.36	<0.001	0.993 (−0.002)	0.992	0.048 (0.003)	0.023 (0.017–0.029) (0.003)
Model 3: Scalar Invariance	334.833 (252)	1.33	<0.001	0.993 (0.000)	0.992	0.046 (−0.002)	0.022 (0.015–0.028) (−0.001)
Model 4: Strict Invariance	347.480 (269)	1.29	<0.001	0.993 (0.000)	0.993	0.047 (0.001)	0.021 (0.014–0.027) (−0.001)

*Note.* X^2^  =  Chi Squared; df = Degree of Freedom; CFI = Comparative Fit Index; TLI = Tucker–Lewis Index; SRMR = Standardized Root–Mean–Square; RMSEA = Root–Mean–Square Error of Approximation; 90% CI = 90% Confidence Interval; ΔCFI = Change in Comparative Fit Index, ΔRMSEA = Change in Root–Mean–Square Error of Approximation.

## Data Availability

The data presented in this study are available upon request to pj.conesacervera@um.es (P.J.C.). The data are not publicly available due to the potential classification as personal health data.

## Appendix A

### Appendix A.1. The Basic Psychological Need Satisfaction in Classroom Scale

#### Appendix A.1.1. Autonomy Need Satisfaction

Q1. Las actividades que hago en clase se ajustan a mis intereses (I feel I have been doing what really interests me in class).

Q5. Las actividades que hago en clase coinciden perfectamente con la forma en que yo quiero hacerlas. (The activities I do in class match perfectly with the way I want to do them).

Q9. La forma de realizar las actividades responde a mis deseos (I feel my choices express who I really am in class).

Q14. Tengo la oportunidad de elegir cómo realizar las actividades (In my class, I feel a sense of choice and freedom in the things I undertake).

#### Appendix A.1.2. Competence Need Satisfaction

Q2. Me siento capaz de alcanzar mis metas en clase (I feel competent to achieve my goals).

Q6. Realizo las actividades de forma eficaz (I am capable of effectively doing even the tasks considered difficult by most of my peers).

Q10. En clase, siento que puedo realizar bien las actividades. (In class, I feel confident that I can do things well).

Q15. Pienso que puedo cumplir con las exigencias de clase (I think I can meet the demands of the class).

#### Appendix A.1.3. Relatedness Need Satisfaction

Q3. Me siento muy cómodo/a con mis profesores y compañeros/as (I feel very comfortable with my teachers and classmates).

Q7. Me llevo bien con mis profesores y compañeros de clase (I feel like I have a close relationship with my teachers and classmates).

Q12. Los profesores y compañeros de clase se preocupan por mí y yo me preocupo por ellos (I feel connected with people who care about me, and for whom I care)

Q16. Me gustan mucho mis profesores y compañeros de clase (I like my teachers and classmates very much).

#### Appendix A.1.4. Novelty Need Satisfaction

Q4. Siento que hago cosas novedosas en clase. (I feel like I’m doing new things in class).

Q8. Siento que a menudo hay novedades para mí, en clase. (I often feel that there is something new for me).

Q11. Experimento sensaciones nuevas. (I feel new sensations in my class).

Q13. Creo que se plantean situaciones novedosas para mí, en clase. (I think new situations are coming for me in class).

Q17. Creo que descubro cosas nuevas a menudo, en clase (I think I often discover new things in class).
